# Development of albumin macroinitiator for polymers to use in DNA origami coating

**DOI:** 10.3906/sag-2001-245

**Published:** 2020-08-26

**Authors:** Aykut BİLİR, Ezgi EMÜL, Necdet SAĞLAM

**Affiliations:** 1 Department of Nanotechnology and Nanomedicine, Graduate School of Science and Engineering, Hacettepe University, Ankara Turkey

**Keywords:** DNA origami, bionanotechnology, atom transfer radical polymerization, drug delivery

## Abstract

**Background/aim:**

DNA nanostructures have many advantages over polymers and lipid based drug delivery agents such as biodegradability and biocompatibility. However their transfection rates and stability still limit their widely use in nanomedicine. In this study highly versatile and straightforward albumin coating preparation method is showed for DNA nanostructures.

**Materials and methods:**

N-methylolmaleimide was esterified with a-bromoisobutyrl bromide (BiBB) to achive bromine functional structure. Then it was attached to bovine serum albumin (BSA) via cysteine-maleimide bond further to use as macroinitiator for atom transfer radical polymerization (ATRP). Cationic polymers can be synthesized from this end further to use as binding domain for fabricated 60 Helix bundle DNA origami.

**Results:**

Proton nuclear magnetic resonance (1H NMR) analysis used for characterization. Methyelene group hydrogens’ peak in 5.0 ppm and strong peak in 1.5–2.0 ppm range showed proper methylolation of maleimide and bromine functional formation, respectively. Then BSA-macroinitiator formation is verified by 1780 Da peak shift in MALDI-TOF (Matrix-assisted laser desorption/ionization - time of flight) spectrum. Moreover electrophoretic mobility shift assay (EMSA) showed successful dense 60 Helix bundle formation.

**Conclusion:**

In this study, a facile method is developed to synthesize protein conjugated-ATRP initiator further can be used in polymerization and coating DNA nanostructures. It is feasible for any protein containing cysteine amino acid.

## 1. Introduction

Structural DNA nanotechnology has made considerable development since Seeman proposed using DNA as a building material for bottom-up self-assembly in 1982 [1], advanced drug delivery [8], synthetic ion channels [9], molecular size electronic circuits [10] and plasmonic nanostructures [11]. 

In addition, these structures are highly suitable for nanomedicine applications thanks to their size (10–100nm). They are inherently biocompatible, nontoxic, biodegradable, have low immunogenicity and can enter cells without transfection agents [12–17]. Numerous smart drugs carriers have developed so far to deliver molecules include fluorescent dyes [18], anticancer drugs [19, 20], CpG (unmethylated cytosine-phosphate-guanine dinucleotides) [14], siRNA [21], enzymes [22]. Despite these unique properties of DNA nanostructures, there are still 2 key challenges to overcome the limiting factors of their extensive usage. Firstly, due to negatively charged hydrophilic nature of DNA origami it shows low cell-transfection rates [23]. Secondly, these structures are often sensitive to cellular medium and depletion of salt ions; although they are slightly stable in cell lysates [24] and can resist nuclease action [25], an efficient method to improve their stability in vivo is urgently needed. 

In order to increase the stability and transfection rates of DNA nanostructures, various materials and protection techniques suggested such as liposome encapsulation [26] and polymer coating [27–31], backbone modification [32], spermidine stabilization [33]. It has been shown that transfection rates can be improved by utilizing DNA intercalators as surface modification agents [34], virus protein [35] and specific proteins studied so far [36]. However, taking into account the accessibility, selectivity and immune responseserum albumin is an easily accessible candidate for coating. Serum albumin is the most abundant blood protein, and has long serum half-life and widely used in clinically approved applications in drug delivery [37,38]. The thiol-maleimide chemistry is frequently used for specific conjugation utilizing cysteine residue in serum albumin [39–42]. Many techniques used to synthesize polymers with protein reactive groups [43–45]. Grafting from approach is the more recent method for synthesizing polymeric biomaterials [46]. By creating specific sites on biomacromolecules, living radical polymerization can be initiated and broad range of protein-polymer conjugates can be achieved. Consequently, by coating DNA nanostructures with serum albumin, can these structures’ stability and transfection rates to the cells be increased? 

In this study, BSA transformed into macroinitiator for atom transfer radical polymerization (ATRP) by targeting the free cysteine residue as reactive towards the maleimide. This initiator can be used for having cationic polymer and utilized as a binding domain that can be further attached to the negatively charged DNA origami surface. It is believed that this method will broaden applications of DNA origami in nanomedicine as a novel alternative to the nanoparticle and lipid based drug delivery carriers.

## 2. Materials and methods

In this study by coating DNA nanostructures with cationic polymer armed albumin protein it is expected to increase these structures’ stability and transfection rates to the cells. The procedure is as follows: using maleimide as a starting material and modifying it to have hydroxyl group. Then from this functional group, a-bromoisobutyrl bromide covalently bonded by esterification. After that bromine functional maleimide conjugated with bovine serum albumin (BSA) by cysteine-maleimide bond. Then ATRP was performed from this macroinitiator by using 2-(dimethylamino) ethyl methacrylate (DMAEMA) as monomer. 60 Helix bundle DNA origami prepared by thermally annealing the scaffold by 141 distinct short staple strands. 

Maleimide supplied from Alfa Aesar [Thermo Fisher (Kandel) GmbH, Kandel, Germany]. Formaldehyde, α-bromoisobutyrl bromide (BiBB), bovine serum albumin, 2-(dimethylamino) ethyl methacrylate (DMAEMA) monomer, ethidium bromide (EthBr) and agarose were purchased from Sigma-Aldrich Chemie GmbH (Taufkirchen, Germany). Milli-Q purified water was used. Scaffold for 60 Helix bundle DNA origami was purchased from TilibitNanosystems (München, Germany) and staple strands from Integrated DNA Technologies Inc. (Coralville, IA, USA). 10x TAE buffer supplied from USB Corporation (Cleveland, OH, USA). Proton nuclear magnetic resonance (1H NMR) spectra were recorded with Bruker Avance 400 MHz (Bruker BioSpin Corporation, Billerica, MA, USA). The chemical shift calibration performed using residual CDCl3 peaks. MALDI-TOF analyses were performed with an UltrafleXtreme 2000 Hz (Bruker Daltonics, Bremen, Germany) with a SmartBeam II laser (355 nm) and operated in positive mode.

### 2.1. Synthesis of N-substituted maleimide derivative

**Figure 1:**
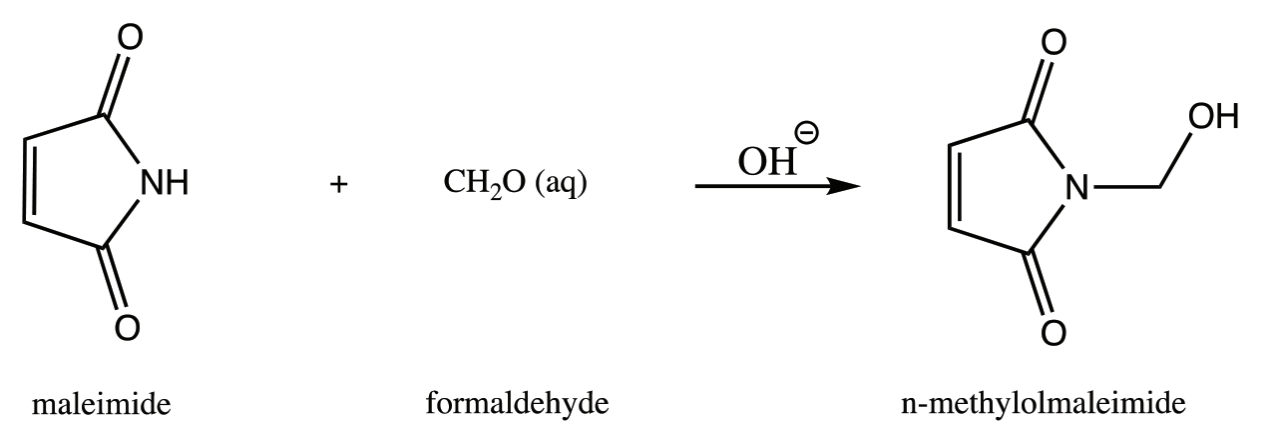
Methylolation of maleimide.

N-methylolmaleimide was synthesized according to the method Tawney et al. proposed (Figure 1) [47]. At room temperature 90 mL of 5% sodium hydroxide was added to a suspension of 2.910 g of maleimide in 5.559 g of 37% formaldehyde (final pH » 5). Within 10 min all of maleimide was dissolved and a mildly exothermic reaction occurred. Separation of the product was begun promptly. After leaving for 2.5 h at room temperature, the solution was filtered. One recrystallization from ethyl acetate ended 1.12 g of product and it was dried under vacuum to remove solvent.

**Figure 2:**
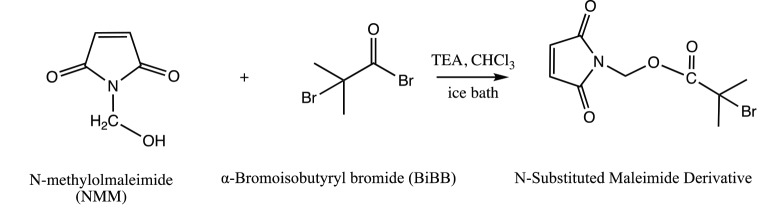
Esterification of N-methylolmaleimide with BiBB.

Bromine functional maleimide was prepared by esterification of N-methylolmaleimide with a-bromoisobutyrl bromide according to the method of Çakır et al. (Figure 2) [48]. Under nitrogen, 1.24 mL (2.307 g, 10 mmol) a-bromoisobutyrl bromide was added dropwise to a stirring mixture of NMM (1 g, 8 mmol) and triethylamine (1.37 mL, 10 mmol) in 23.3 mL of CHCl3 in an ice bath for 1 h. After complete addition of the acid bromide, the reaction was stirred at room temperature for 3 h. A dark red reaction mixture observed and it was washed with water (3 × 23.3 mL) and then dried over MgSO4. After filtration and evaporation of CHCl3, it was dried under vacuum and recrystallized from ethanol.

### 2.2. BSA-macroinitiator

**Figure 3:**
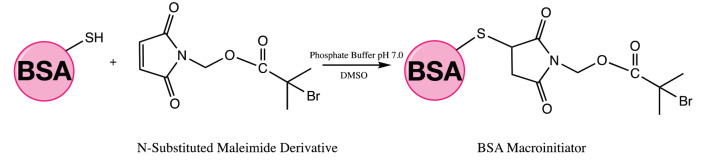
Thioether bond formation between cysteine-maleimide derivative.

Synthesis of BSA-macroinitiator targeted the free cysteine residue (Cys-34) as reactive towards the maleimide, described earlier by Nicolas et al. (Figure 3) [49]. BSA (34 mg, 0.52 μmol) was dissolved in 4.6 mL of 100 mM phosphate buffer (pH = 7.0). A solution of 12.2 mg of N-substituted maleimide derivative in 0.2 mL of DMSO was added to this slowly. The mixture was gently stirred during 24 h at ambient temperature and the solid residue was then removed by centrifugation. The supernatant was diluted with deionized water and dialyzed with a 12,000–14,000 Da molecular weight cut-off (MWCO) membrane against deionized water for 3 days. The solution was then lyophilized to isolate the BSA-macroinitiator. 

### 2.3. Atom transfer radical polymerization (ATRP)

**Figure 4:**
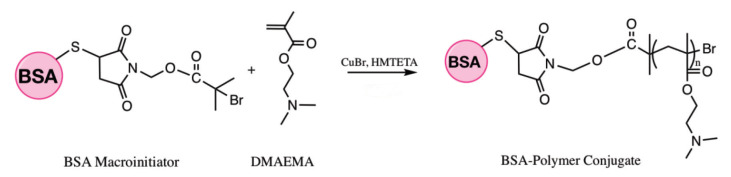
ATRP of BSA-polymer conjugate.

BSA-polymer conjugate achieved via ATRP from BSA macroinitiator, by using modified method of Välimäki et al. (Figure 4) [50]. CuBr (53 mg, 0.37 mmol) was placed in a round-bottom flask with a stirring bar. BSA macroinitiator (25 mg, 0.37 mmol) and ligand HMTETA (170 mg, 0.74 mmol) dissolved in 10 mL of water and purged with nitrogen gas for 15 min. Then the solution transferred to the flask by cannula. The same procedure repeated for DMAEMA (5.8 g, 37 mmol) and the polymerization was performed for 4 h in a sealed flask at room temperature with magnetic stirring. For 240 min, every 30 min sample was taken from the flask to vials, then they were opened to air and placed in an ice bath to stop the reaction. The solid residue was removed by centrifugation, and the supernatant was lyophilized to give blue product.

### 2.4. Preparation of DNA origami nanostructure 

60 Helix bundle DNA origami nanostructure was prepared, purified and characterized as described earlier [51,52]. Briefly, folding solution obtained by mixing following substances:

20 mL M13mp18 scaffold (100 nM)

40 mL staples (500 nM)

40 mL folding buffer containing 2.5 TAE, 12.5 mM NaCl and 50 mM MgCl2

Then final solution was annealed by G-Storm thermocycler (Gene Technologies Ltd., Melbourne, Australia). The excess amount of staples was removed by PEG-based purification. Folding and purification of DNA origami is characterized by agarose gel electrophoresis. 

## 3. Results and discussion

**Figure 5:**
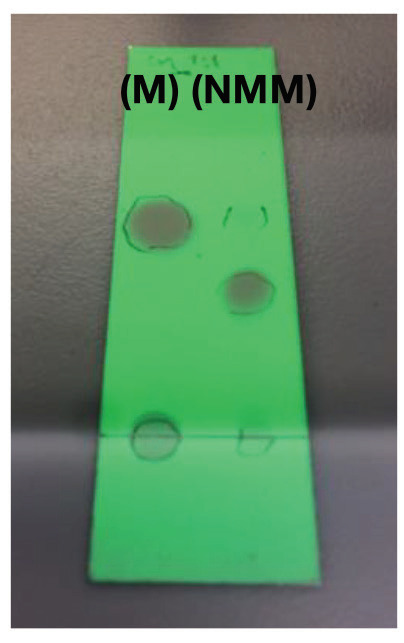
Thin layer chromatography of maleimide and N-methylolmaleimide.

**Figure 6:**
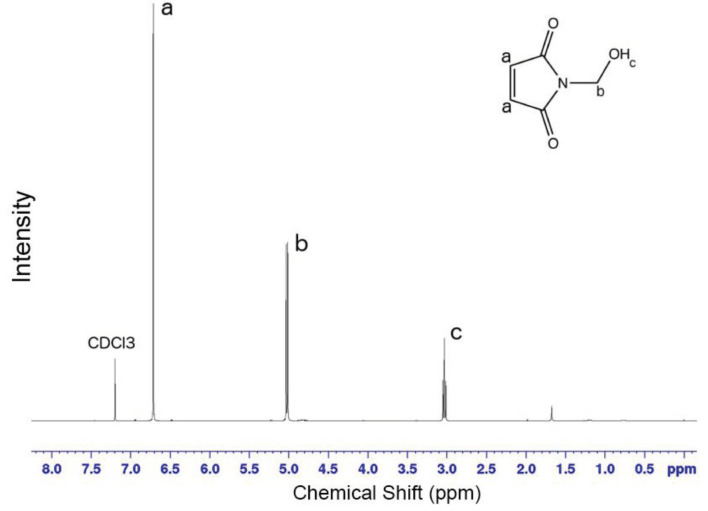
1H NMR spectrum of N-methylolmaleimide.

**Figure 7:**
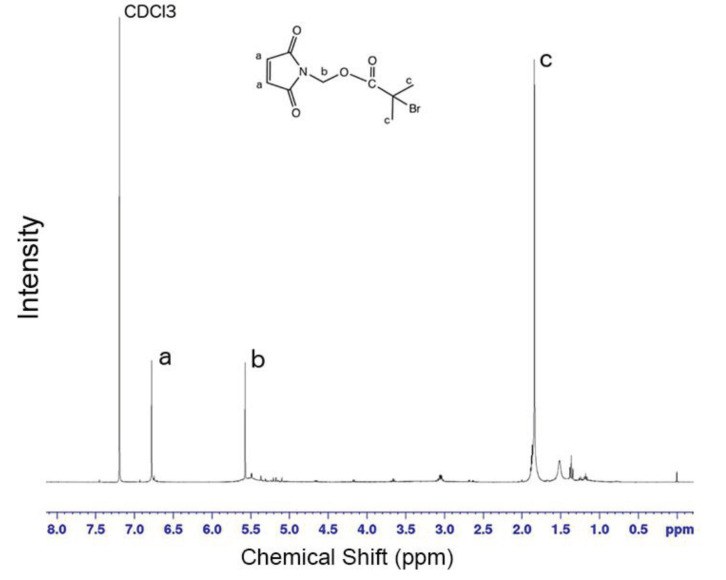
1H NMR spectrum of bromine functional maleimide (MBr).

**Figure 8:**
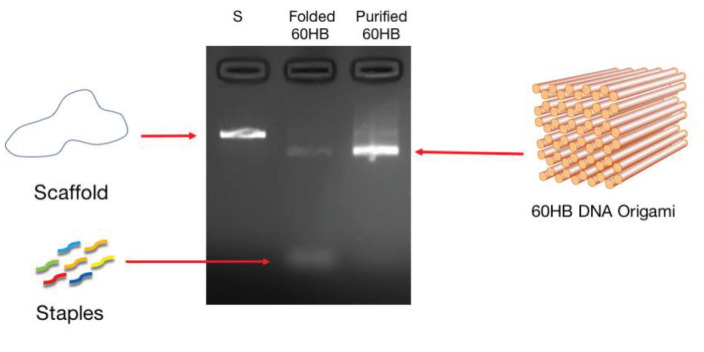
MALDI-TOF spectra of A) BSA and B) BSA macroinitiator.

**Figure 9:**
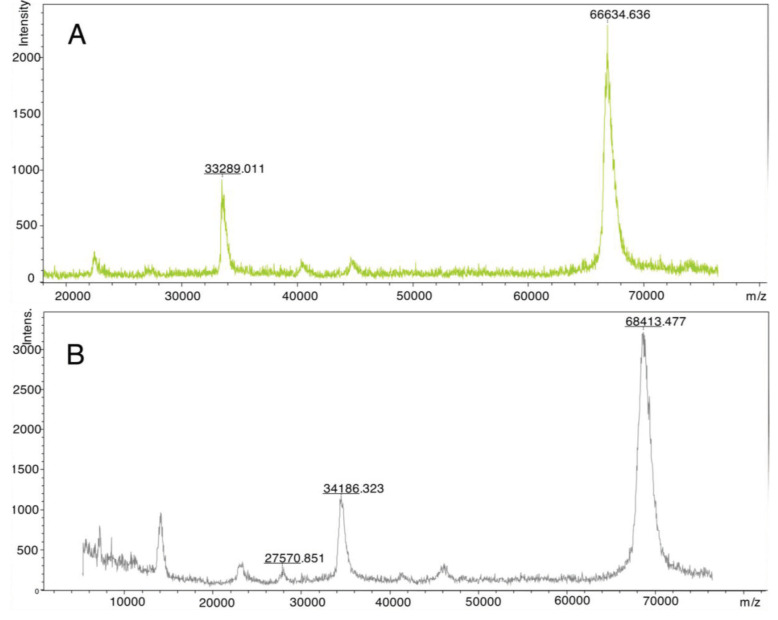
Resonance signals of according to chemical shifts shown in ppm in NMR spectra of samples taken from polymerization medium after A) 30 min, B) 240 min C) 240 min and dilution with water 1:1 volumetric ratio.

**Figure 10:**
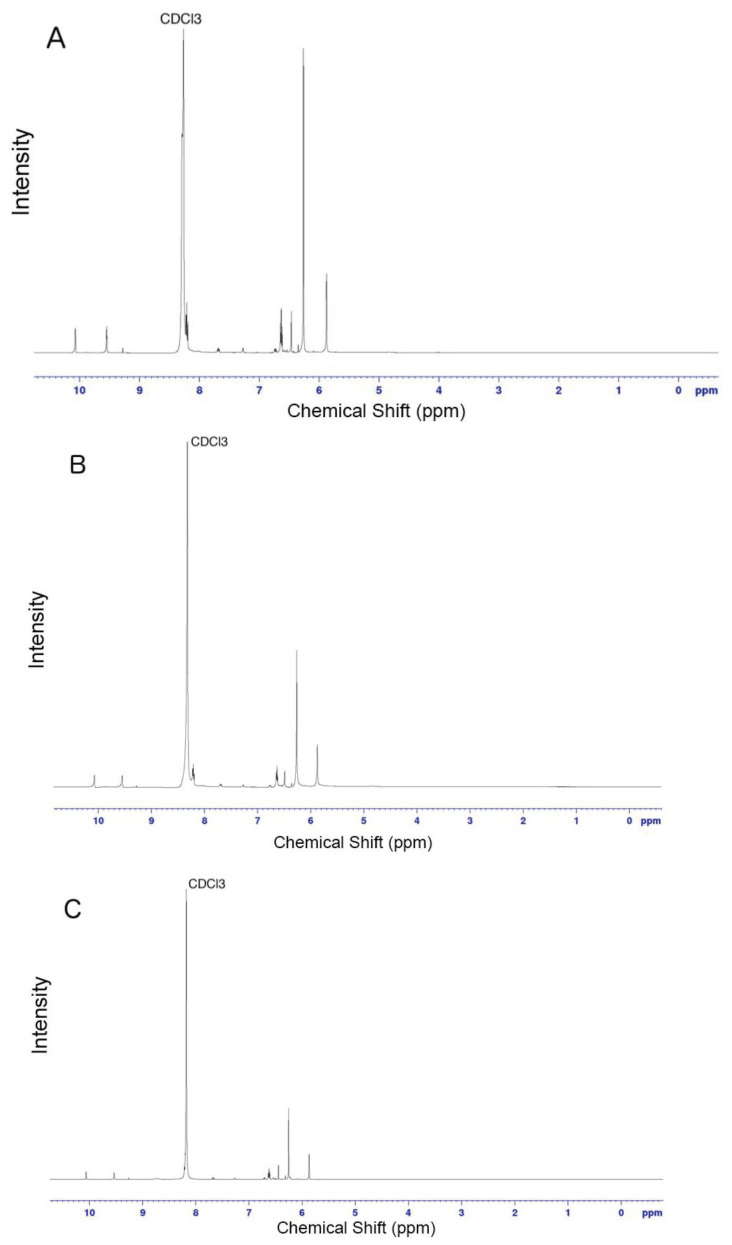
Electrophoretic mobility shift assay (EMSA) results for folded and purified 60HB DNA origami.

Similar results observed with the literature in the methylolation of maleimide, maleimide quickly dissolved, and gave an exothermic reaction [47]. Thin layer chromatography of maleimide and methylolation step product confirmed the proper methylolation of maleimide. Maleimide runs faster than N-methylolmaleimide and was observed under UV light (Figure 5).The structure of N-methylolmaleimide was confirmed by 1H NMR recorded in CDCl_3_ as well. 1H NMR spectrum displays signals corresponding to hydrogens of methylene group and hydroxyl group thus proves the proper methylolation of maleimide (Figure 6). 1H NMR (CDCl_3_), d (ppm): 3.01–3.05 (t, 1H), 5.00–5.04 (d, 2H), 6.70–6.72 (s, 2H). To validate the structure of bromine functional maleimide (MBr), 1H NMR spectrum recorded in CDCl3. The resonance signals and relative intensities corresponding to methylene (-CH_2_) and methyl (-CH_3_) groups evidenced successful esterification, denoted in b and c respectively and it is found compatible with the results reported earlier (Figure 7) [48]. 1H NMR (CDCl3), d (ppm): 1.82–1.85 (s, 6H), 5.56–5.57 (s, 2H), 6.77–6.78 (s, 2H). Conjugation of bromine functional maleimide (MBr) to BSA was demonstrated by MALDI-TOF. Incubation of BSA with MBr showed a shift to position 68413 m/z (Figures 8A and 8B). This corresponds to a change of 1780 Da and proves proper conjugation of bromine functional maleimide (MBr) to BSA (8). Polymerization from BSA macroinitiator was followed by 1H NMR. In order to be ensure the complete dissolution of the sample, 0.7 mL CDCl3 mixed with 0.3 mL CD3OD and used as solvent. The resonance signal corresponding to repeating unit (methylene) group of PDMAEMA was searched around 1.0 ppm, however no peak observed in after 30 min (Figure 9A), 240 min. (Figure 9B), 240 min and dilution with water 1:1 volumetric ratio (Figure 9C). This can be attributed to side reaction between functional groups on amino acid residues and DMAEMA monomer. Moreover, the mechanism can be slow due to the big structure of BSA, and 240 min is not enough to observe the polymer peak on 1H NMR spectrum. To overcome this issue, different polymerization medium and ratios of the substances will be tried. Gel electrophoretic mobility shift assay (EMSA) verified that folded and compact 60HB runs faster than the scaffold as shown in (Figure 10). Besides, PEG purification step removed the excess staples efficiently. However there may be some possible limitations in the study such asin MALDI-TOF analysis chemical shift between BSA and BSA macroinitiator comparison, it is unclear the ratio of one to one chemical conjugates to the electrostatically gathered structures.

## 4. Conclusion

As a conclusion, we demonstrated a facile and widely applicable method to prepare BSA macroinitiator that can be further used in preparation of polymeric biomaterials. Characterization stages have given valuable information about transformation of maleimide to bromine functional initiator and thioether formation by Michael addition of maleimide end to thiol of cysteine residues. Though the weak polymer peak for backbone in 1H NMR spectrum, a clear shift in MALDI-TOF spectra has provided an evidence for protein-initiator binding. In this method, any cysteine containing proteins can be selectively conjugated with bromine functional ATRP initiator, however polymerization medium should be taken into account to avoid denaturation of protein. Therefore, this approach offers notable opportunities which can be extended not only for DNA origami coating but also for responsive biohybrid material development. This method makes considerable contribution for attracting the attention to DNA origami studies and to future studies with BSA-like biocompatible proteins and polymers.

## Acknowledgments/disclaimers

Aykut Bilir gratefully acknowledge Finnish National Agency for Education for their scholarship. Ezgi Emül. and Aykut Bilir thank Turkish Council of Higher Education for scholarship. The work was carried out under the Academy of Finland’s Centres of Excellence Programme (2014–2019). The authors wish to thank Biohybrid Materials Group members at Aalto University, SallaVälimäki for help during macroinitiator synthesis, HeiniIjäsfor characterization in DNA origami studies and Dr. Mauri A. Kostiainen for helpful discussions. 

## Conflict of interest

This study was presented at the Taiwan-Turkey Science Summit entitled “Translation of Cells, Nanomaterials and Signaling Molecules into Regenerative Medicine” between April 1 to 3, 2018.

## References

[ref1] (1982). Nucleic acid junctions and lattices. Journal of Theoretical Biology.

[ref2] (2011). DNA origami with complex curvatures in three-dimensional space. Science.

[ref3] (2009). Self-assembly of DNA into nanoscale three-dimensional shapes. Nature.

[ref4] (2009). Self-assembly of a nanoscale DNA box with a controllable lid. Nature.

[ref5] (2006). Folding DNA to create nanoscale shapes and patterns. Nature.

[ref6] (2019). Facile and label-free electrochemical biosensors for microRNA detection based on DNA origami nanostructures. ACS Omega.

[ref7] (2019). Precise regulation of enzyme cascade catalytic efficiency with DNA tetrahedron as scaffold for ultrasensitive electrochemical detection of DNA. Analytical Chemistry.

[ref8] (2012). A logic-gated nanorobot for targeted transport of molecular payloads. Science.

[ref9] (2012). Synthetic lipid membrane channels formed by designed DNA nanostructures. Science.

[ref10] (2009). Characterization of the conductance mechanisms of DNA origami by AC impedance spectroscopy. Small.

[ref11] (2012). DNA-based self-assembly of chiral plasmonic nanostructures with tailored optical response. Nature.

[ref12] (2012). Correction to rolling circle amplification-templated DNA nanotubes show increased stability and cell penetration ability. Journal of the American Chemical Society.

[ref13] (2019). Rationally designed DNA-origami nanomaterials for drug delivery in vivo. Advanced Materials.

[ref14] (2011). Self-assembled multivalent DNA nanostructures for noninvasive intracellular delivery of immunostimulatory CpG oligonucleotides. ACS Nano.

[ref15] (2017). DNA origami applications in cancer therapy. Cancer Science.

[ref16] (2011). DNA cage delivery to mammalian cells. ACS Nano.

[ref17] (2017). Sequence-independent DNA nanogel as a potential drug carrier. Macromolecular Rapid Communications.

[ref18] (2014). DNA nanoflowers for multiplexed cellular imaging and traceable targeted drug delivery. Angewandte Chemie International Edition.

[ref19] (2012). DNA origami as a carrier for circumvention of drug resistance. Journal of the American Chemical Society.

[ref20] (2012). DNA origami delivery system for cancer therapy with tunable release properties. ACS Nano.

[ref21] (2012). Molecularly self-assembled nucleic acid nanoparticles for targeted in vivo siRNA delivery. Nature Nanotechnology.

[ref22] (2016). Cellular delivery of enzyme-loaded DNA origami. Chemical Communications.

[ref23] (2014). Quantification of cellular uptake of DNA nanostructures by qPCR. Methods.

[ref24] (2011). Stability of DNA origami nanoarrays in cell lysate. Nano Letters.

[ref25] (2011). A primer to scaffolded DNA origami. Nature Methods.

[ref26] (2014). Virus-inspired membrane encapsulation of DNA nanostructures to achieve in vivo stability. ACS Nano.

[ref27] (2017). Oligolysine-based coating protects DNA nanostructures from low-salt denaturation and nuclease degradation. Nature Communications.

[ref28] (2016). Cationic polymers for DNA origami coating - examining their binding efficiency and tuning the enzymatic reaction rates. Nanoscale.

[ref29] (2017). Cuboid Vesicles Formed by Frame-Guided Assembly on DNA Origami Scaffolds. Angewandte Chemie-International Edition.

[ref30] (2018). (Poly)cation-induced protection of conventional and wireframe DNA origami nanostructures. Nanoscale.

[ref31] (2017). Block copolymer micellization as a protection strategy for DNA origami. Angewandte Chemie-International Edition.

[ref32] (2017). Enhanced stability of DNA nanostructures by incorporation of unnatural base pairs. Chemphyschem.

[ref33] (2016). Electrotransfection of polyamine folded DNA origami structures. Nano Letters.

[ref34] (2015). Designed intercalators for modification of DNA origami surface properties. Chemistry.

[ref35] (2014). Virus-encapsulated DNA origami nanostructures for cellular delivery. Nano Letters.

[ref36] (2016). Intracellular delivery of a planar DNA origami structure by the transferrin-receptor internalization pathway. Small.

[ref37] (2018). Porphyrinoid biohybrid materials as an emerging toolbox for biomedical light management. Chemical Society Reviews.

[ref38] (2016). Synergistic effect of human serum albumin and fullerene on Gd-DO3A for tumor-targeting imaging. ACS Applied Materials & Interfaces.

[ref39] (1999). Therapeutic antibody fragments with prolonged in vivo half-lives. Nature Biotechnology.

[ref40] (2004). Effects of drug loading on the antitumor activity of a monoclonal antibody drug conjugate. Clinical Cancer Research.

[ref41] (2008). Site-specific conjugation of a cytotoxic drug to an antibody improves the therapeutic index. Nature Biotechnology.

[ref42] (2009). Antibody-drug conjugates for the treatment of non-Hodgkin’s lymphoma: target and linker-drug selection. Cancer Research.

[ref43] (2007). Synthesis of protein-polymer conjugates. Organic & Biomolecular Chemistry.

[ref44] (2007). Living radical polymerization as a tool for the synthesis of polymer-protein/peptide bioconjugates. Macromolecular Rapid Communications.

[ref45] (2008). Straightforward synthesis of cysteine-reactive telechelic polystyrene. Macromolecules.

[ref46] (2015). Smart hybrid materials by conjugation of responsive polymers to biomacromolecules. Nature Materials.

[ref47] (1960). The chemistry of maleimide and its derivatives. I. N-Carbamylmaleimide. The Journal of Organic Chemistry.

[ref48] (2006). Graft copolymerization of methylmethacrylate with N-substituted maleimide-styrene copolymer by ATRP. Journal of Applied Polymer Science.

[ref49] (2006). Fluorescently tagged polymer bioconjugates from protein derived macroinitiators. Chemical Communications.

[ref50] (2016). Effect of PEG-PDMAEMA block copolymer architecture on polyelectrolyte complex formation with heparin. Biomacromolecules.

[ref51] (2017). Protein coating of DNA nanostructures for enhanced stability and immunocompatibility. Advanced Healthcare Materials.

[ref52] (2014). Facile and scalable preparation of pure and dense DNA origami solutions. Angewandte Chemie-International Edition.

